# Letter to the Editor on “From Concept to Clinic: Living Labs and Regulatory Sandboxes for Health System Digitalization and the Integration of Innovative Devices Into Clinical Workflows”

**DOI:** 10.1109/JTEHM.2025.3557508

**Published:** 2025-03-31

**Authors:** Rebecca Mathias, Anett Schönfelder, Cindy Welzel, Stephen Gilbert

**Affiliations:** Else Kröner Fresenius Center for Digital HealthTUD Dresden University of Technology Dresden 01307 Germany

## Abstract

Digital health and AI-enabled technologies hold the promise of addressing gaps in healthcare, but balancing rapid market access with the need for safe, functional, and user-centered solutions remains a challenge [1], [2]. Regulatory requirements for device development and market approval demand detailed documentation and predetermined protocols, which can limit the adaptability developers require for iterative improvement and real-world testing with patients and healthcare professionals [1], [3], [4]—an approach that would be highly beneficial for digital and AI-enabled technologies. As a result, key factors like clinical workflow integration, interoperability, and usability with the real range of in-use devices are often overlooked or addressed in a cursory fashion [5].

Digital health and AI-enabled technologies hold the promise of addressing gaps in healthcare, but balancing rapid market access with the need for safe, functional, and user-centered solutions remains a challenge [1], [2]. Regulatory requirements for device development and market approval demand detailed documentation and predetermined protocols, which can limit the adaptability developers require for iterative improvement and real-world testing with patients and healthcare professionals [1], [3], [4]—an approach that would be highly beneficial for digital and AI-enabled technologies. As a result, key factors like clinical workflow integration, interoperability, and usability with the real range of in-use devices are often overlooked or addressed in a cursory fashion [5].

Living Labs and Regulatory Sandboxes are two related approaches that allow greater freedom of experimentation and hold promise for addressing these challenges by supporting smoother integration into clinical workflows [6] (Figure. 1). Living Labs function as innovation ecosystems where stakeholders co-design and test products in near real-world settings, emphasizing user engagement and iterative development. Similarly, Regulatory Sandboxes provide real-world-inspired environments for testing but focus on collaboration between companies and regulators, allowing temporary regulatory flexibility to assess market feasibility under supervisory oversight. However, current regulations governing clinical development and testing methodologies [7], [8], [9] are not yet fully suited to accommodate these models [1], creating tension between fostering innovation and ensuring compliance. Until regulations evolve, developers must carefully balance agility with compliance with rigid approval regimes.

**FIGURE 1.**
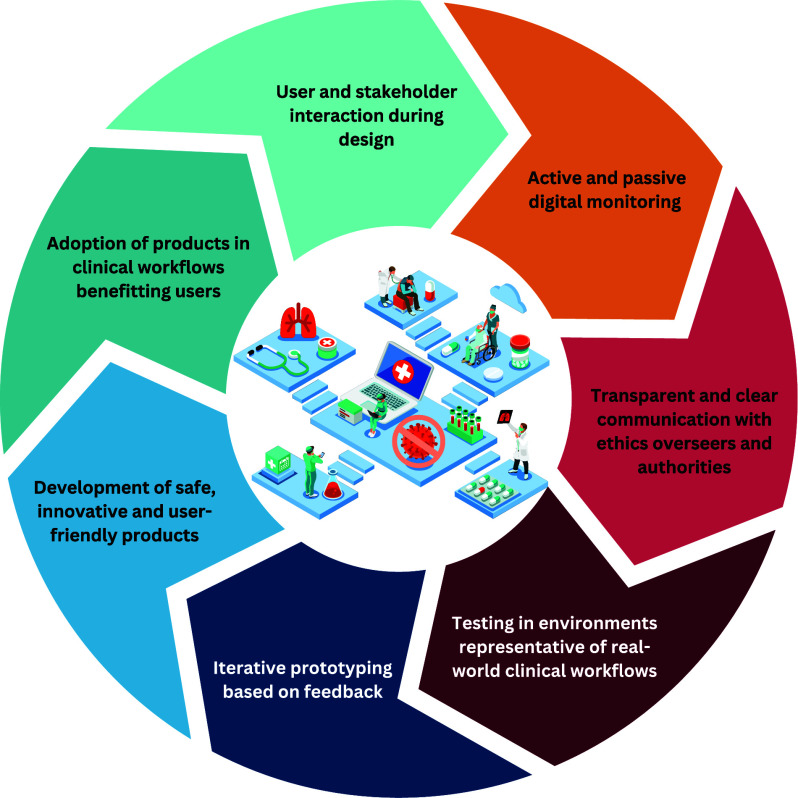
Features of Living Labs and Regulatory Sandboxes that enable stakeholder interaction and real-world feedback in early and late product clinical development and clinical validation. Digitally advanced Living Labs can provide continuous and consented monitoring that enhances clinical insight, that can enable greater flexibility in clinical study design, safe iteration of product design in testing, and that can support the true goals of regulatory approval processes: safe products well-adapted to real-use scenarios and that can be better integrated into clinical workflows.

Advancements in AI-enabled monitoring and digital automation can support adaptive development while maintaining oversight and safety standards. Wearable and contact-free monitoring tools allow continuous data collection, improving decision-making and patient safety while supporting regulatory processes [10]. Integrating this data into Quality Management Systems (QMS) can automate documentation and procedures, streamlining processes while ensuring rigorous oversight. The use of dynamic consent models should also be part of this framework, enabling patients and healthcare professionals to manage data collection and sharing in real time [11].

Predetermined Change Control Plans (PCCPs), originally designed for market approval, could also be applied to testing methodologies by defining acceptable changes in device performance without requiring repeated formal amendments [12], [13]. Clear and concise standard operating procedures (SOPs) and study plans for Living Labs can fix boundaries for experimentation without stifling innovation, ensuring regulatory integrity while allowing flexibility.

Due to a lack of practical approaches, perceived high costs [14], and inadequate governance frameworks, Living Labs are not yet fully standardized within healthcare strategies. However, regulatory thinking is evolving. For example, in the USA, the FDA has introduced the “Home as a Health Care Hub” initiative, aiming to incorporate system-level and user-centric approaches into digital health innovation [15]. In the EU, the AI Act requires member states to establish Regulatory Sandboxes for AI by 2026 [16]. These Sandboxes currently focus more on late-stage development than early innovation though, and their implementation details remain unclear.

We argue that in order to realize the vision for safe and innovative digital health that significant investment in Regulatory Sandboxes is essential. These Sandboxes must operate in a flexible, user-centric, real-world testing manner—similar to Living Labs, exploring not just one medical device under development, but the changes in hospital workflows and healthcare provider roles that accompany innovation, and particularly digital transformation. A risk of stringent regulations applied without flexibility or pragmatism is the discouragement of the development of devices that can function well together and be adaptable to clinic needs. An important example of past failure in this regard is the disruption, stress, and fatigue of healthcare providers through multiple incompatible and noninteroperable medical device alarm systems, designed for the liability safeguarding of the manufacturer rather than for the true safeguarding of the patient [17]. It is imperative that similar problems are avoided in future digital transformation and the implementation of AI in medicine.

Without adequate investment in Regulatory Sandboxes, operating as Living Labs, we risk stifling innovation, losing market interest, and missing out on healthcare solutions that are patient-centered, technically sound, and grounded in strong safety frameworks.
